# Health-related quality of life and cognitive emotion regulation strategies in the unemployed: a cross-sectional survey

**DOI:** 10.1186/s12955-014-0172-6

**Published:** 2014-11-29

**Authors:** Natalio Extremera, Lourdes Rey

**Affiliations:** Department of Social Psychology, Faculty of Psychology, University of Malaga, Campus de Teatinos s/n. Málaga, 29071 Malaga, Spain; Department of Personality, Evaluation and Psychological Treatment, University of Malaga, Malaga, Spain

**Keywords:** Quality of life, Unemployment, Cognitive coping, Emotion regulation strategies

## Abstract

**Background:**

The loss of one’s job has been conceptualized as a major stressful life event in an adult’s life and has consistently been associated with lower health-related quality of life (HRQoL). The role of cognitive emotion regulation strategies after the experience of stressful events has emerged as an important predictor of adverse psychological and health outcomes. However, the effect of these coping strategies on the HRQoL of unemployed people has not been examined until now. We aimed to study the associations of these cognitive emotion regulation strategies on HRQoL of unemployed people.

**Methods:**

Using cross-sectional data, 1,125 unemployed adults were assessed using a Cognitive Emotion Regulation Questionnaire for cognitive coping and SF-12 to assess HRQoL. We studied the effect of cognitive emotion regulation strategies on mental and physical health composite separately, adjusting for gender, age, educational level and length of unemployment, using hierarchical regression analyses.

**Results:**

Results showed unemployed men tended to express greater use of self-blame, other-blame, and catastrophizing, and lower use of perspective taking strategies when confronted with unemployment. Moreover, self-blame (for mental health composite only), blaming others, rumination, and catastrophizing negatively correlated, while positive reappraisal, putting into perspective, planning, and positive refocusing positively correlated with both mental and physical health composite in unemployed people. Further hierarchical regression analyses indicate that five strategies (a lower reported use of self-blame, rumination, and catastrophizing, as well as higher scores of positive reappraisal and positive refocusing) and three strategies (lower scores of catastrophizing and rumination and high scores in planning) have significant and independent contributions, beyond gender, age, educational level and length of unemployment, to the prediction of mental health and physical health composite, respectively.

**Conclusions:**

Cognitive emotion regulation strategies appeared to be significantly correlated with HRQoL in unemployed people. Our findings suggest the potential value of including assistance programs focused on cognitive emotion regulation strategies to complement current psychosocial and health approaches aimed at preserving or improving unemployed people’s HRQoL.

## Background

Unemployment remains a major social problem in most European countries, including Spain. At the time of this study, the national unemployment rate in the first quarter of 2014 for Spain was 25.93% with 5,933,000 individuals unemployed [1]. The loss of one’s job has consistently been associated with adverse health and psychological outcomes for individuals, their families, and society at large [2,3]. Unemployment has also been associated with deleterious health problems such as depression or psychopathologies [4], low level of self-esteem [5], and with a significant increase in suicide rates [6,7]. The change in social status, time-structure, disruptions in both work and family roles, loss of self-concept and identity, decreasing social contacts, subsequent financial deprivation, and uncertainty about the future have been attributed to be possible sources of pervasive adverse consequences for health-related quality of life (HRQoL) [8]. In this respect, identifying psychosocial moderator variables within a stressful life event perspective, that either protect people or place them at greater risk for the consequences of job loss, remains an important focus for both career counsellors and social researchers [9]. Despite similar financial or family conditions, some unemployed people may develop more problems in quality of life than others [2]. A growing body of evidence has consistently demonstrated that health problems and emotional distress in response to a life stressor may be associated with personal coping strategies that individuals use to deal with job loss and unemployment [10]. A series of studies have revealed that coping styles related to the way people think and feel about the implications of unemployment and how they can minimize the personal, social, and economic threat of this situation might be important predictors of subsequent emotional distress and psychological outcomes [11]. For example, appraisal of job loss as a negative event has been associated with poor mental health and internal attributions for job loss have been significantly related to higher physical health. In contrast, exerting effort in a job search has been associated with higher mental health during unemployment. In sum, the style of coping that unemployed people use to deal with psychological distress during unemployment seems to be associated, to some degree, with their levels of physical and mental health [12].

The current framework and research on coping rests on the notion that coping primarily involves conscious strategies of responding to stressful negative events [13]. Although individuals vary in how they deal with job loss [14,15], coping is typically classified into two general categories: emotion-behavioral coping and cognitive focused coping [16,17]. Whereas emotion-related behavior coping refer to attempts to act on the stressor, emotion-focused cognitive coping refers to attempts to manage the emotions associated with the stressor [18]. One issue arising in the literature on coping refers to the fact that both problem and emotion-focused dimensions are made up of a mix of cognitive and behavioral coping strategies (i.e. thinking and acting), while it would be reasonable to assume that, theoretically, cognitive coping and behavioral actions can be considered as very different processes [19–21]. Garnefski and colleagues suggest that, in order to make a significant contribution to the coping field, it would be necessary to focus on specific and conceptually pure coping subtypes. In fact, in a valuable meta-analytic study on psychological and physical health during unemployment, the authors concluded that findings in unemployment fall across different forms of problem-focused (i.e., seeking retraining and seeking relocation are considered together) and emotion-focused coping (i.e., distancing from loss and seeking social support are considered together) because of a lack of studies focused on coping strategies in the unemployment literature [2]. In the present study cognitive emotion regulation strategies will therefore be studied as a cognitive way of handling the intake of emotionally arousing information [22], separate from behavioral strategies. This refers to the part of emotion regulation concerning the conscious, cognitive processes by which individuals regulate their own emotions [20].

Garnefski and colleagues have developed a conceptual approach of coping strategies called cognitive emotion regulation strategies [20–23]. These strategies mainly involve the conscious, relatively psychological styles individuals use to deal with negative events in their lives [20]. In certain situations people may use specific cognitive emotion regulation strategies, which may differ from other strategies that could be used in other different contextual situations. Such cognitions and thoughts might be key in their ability to regulate emotions, and not be overwhelmed by them, during stressful life events such as long periods of unemployment.

Several studies have found that these cognitive emotion regulation strategies are associated with adverse health outcomes, including depression, anxiety, or psychological maladjustment [23–25]. Specifically, a number of recent findings, both in the general adult population and in different samples across different types of stressful life event, have revealed that maladaptive cognitive emotion regulation strategies such as self-blame, rumination, and catastrophizing, show strong relations with indicators of emotional problems such as anxiety or depression [20,26–28]. On the contrary, adaptive cognitive emotion regulation strategies, including acceptance, positive refocusing, and refocusing on planning, are central to well-being and successful functioning and organization of adaptive behaviors following disruptive life events [29]. Garnefski and colleagues have considered coping strategies from existing measures, either by removing and reformulating the cognitive dimensions, by adapting non-cognitive coping strategies into cognitive dimensions or by adding new strategies on “rational” grounds, for which they used classic instruments such as Coping Inventory for Stressful Situations (CISS) [30]. High correlations were found between the Refocus on Planning and Positive Reappraisal from CERQ subscales and the CISS Task-oriented Coping subscale. These scales all reflect the active coping with or management of the problem. In addition, Acceptance and Putting into Perspective showed reasonably high correlations with Task-oriented Coping. Besides, high correlations were found between CERQ subscales Self-blame, Rumination and Catastrophizing and the CISS Emotion-oriented Coping subscale, all referring to a certain way of being preoccupied with your emotions and in general considered less functional strategies. In general, results showed that the strongest correlations are found between a number of CERQ scales and the scales of the CISS that are related as regards content, providing significant evidences of construct validity [21].

As far as we know, no studies have tested the specific role that cognitive emotion regulation strategies play in adaptation to the multiple stressors and transitions associated with the situation of unemployment. With an increasing proportion of the population becoming unemployed in nearly all European countries [1,31,32] and a bulk of empirical research demonstrating that most unemployed people experience physical and psychological problems [2,9,33], an understanding of the ways people cope with unemployment is necessary. Such examination of cognitive mechanisms would also help to identify people who suffer less than others do when unemployed, and in so doing would enable us to design effective assistance interventions to prevent the emotional distress of unemployment. In the present study we analyzed the relationship between these cognitive emotion regulation strategies and HRQoL in a sample of unemployed adults. HRQOL is a multidimensional concept that includes physical, psychological and social domains of health and is generally accepted as an important outcome measure of health care, especially during unemployment [8]. A main approach to measuring HRQOL is generic instrument, being the 12-item Health Survey (SF-12) and larger version (SF-36), the most widely used in health status studies involving the general population, as well as in studies with disease groups [34,35].

## Methods

### Participants and procedure

Subjects were approached and asked to volunteer in a study on “well-being and unemployment” in different National Employment agencies in southern Spain. The subjects attending the centers were approached by one/two psychology students and asked to answer some questionnaires anonymously. Further respondents were recruited through a snowball sampling technique via the researchers’ and undergraduates’ personal and professional contacts. Although sampling bias is a possible drawback of this method [36], the instructions on the questionnaires were precise to avoid these biases to a greater extent. After providing oral informed consent, the participants completed the questionnaires at home and returned them to the research assistant. Common inclusion criteria were being unemployed and actively looking for a job at the time of this survey. Exclusion criteria were illiteracy in Spanish and being unemployed but not actively job searching (i.e. medical condition, homemakers, pregnant…). Participants received a questionnaire set containing the measures assembled in this study and additional ones for other scientific purposes. Approximately 70% of those approached were willing to participate. No financial compensation was offered to the subjects for their participation. Overall, 1,125 participants (506 men and 619 women) were included in the data analyses. The mean age was 35.30 years (SD = 11.29), (range 17 to 64 years). The educational level in the present sample was: 6% no studies; 36% primary studies; 17% incomplete secondary; 19.4% complete secondary studies; 19.2% university studies; 2.2% post-graduate studies. The average duration of unemployment was 22.17 months (SD = 30.94 months). 66% had been unemployed for more than 12 months. The marital status of the participants was: 49.9% single, 34.5% married, 8.5% separated/divorced, 1.9% widow/widower and 5% couple. Three subjects did not indicate their marital status. The investigated study sample was quite representative to Spanish general population of unemployed in terms of gender. According to official data of National Statistics Institute, 51.4% of unemployed were males and 48.6% were males (in the study sample – 45% and 55%, respectively). However, the study sample was found to be non-representative of the unemployed in Spain in terms of age, being generally older in our study than in Spanish Statistics [1].

### Measures

Two well-validated questionnaires were used to measure cognitive emotion regulation strategies and HRQoL. All participants had to answer the questionnaires concerning their cognitive emotion regulation strategies and mental and physical quality of life regarding their current unemployment situation. Results showed acceptable Cronbach’s alphas for all the used measures (see Table 1).

**Table 1:** **Partial intercorrelations among cognitive emotion regulation strategies and SF-12 factors**

	**1**	**2**	**3**	**4**	**5**	**6**	**7**	**8**	**9**	**10**	**11**
1. Self-blame	--										
2. Acceptance	.39**	--									
3. Rumination	.41**	.38**	--								
4. Positive Refocusing	.15**	.33**	.20**	--							
5. Refocus on Planning	.19**	.38**	.38**	.55**	--						
6. Positive Reappraisal	.22**	.42**	..24**	.58**	.64**	--					
7. Putting into Perspective	.15**	.42**	.15**	.50**	.50**	.58**	--				
8. Catastrophizing	.34**	.19**	.45**	.05	.08**	-.01	-.04	--			
9. Other-blame	-.05	.05	.18**	.09	.06*	-.08**	.06*	.37**	--		
10. Mental Composite Summary	-.13**	-.01	-.20**	.22**	.07*	.21**	.13**	-.24**	-.08**	--	
11. Physical Composite Summary	-.05	-.05	-.11**	.06*	.13**	.07*	.06*	-.21**	-.10**	.04	--
Alpha	.70	.62	.69	.79	.76	.79	.69	.71	.83	.76	.74

Note:*p < 0.05; **p < 0.01.

### The 12-item short form health survey (SF-12 v.1.0)

The SF-12 is a shorter alternative to the SF-36 Health Survey instrument [34]. The SF-12 is a multipurpose short form survey with 12 questions, all selected from the SF-36 Health Survey. The SF-12 includes 8 concepts commonly represented in health surveys: physical functioning, physical role functioning, bodily pain, general health, vitality, social functioning, emotional role functioning, and mental health. Results are expressed in terms of two total scores: the Mental Component Summary (MCS) and the Physical Component Summary (PCS). We used the well-validated Spanish version of SF-12 [35]. In the present study, according to the test manual, standardized scores (M = 50, SD = 10) were calculated where higher scores indicate better health status.

### Cognitive Emotion Regulation Questionnaire (CERQ)

The CERQ assesses the cognitive emotion regulation strategies of people in response to threatening or stressful life events, in this case being unemployed. The 36-item CERQ contains nine conceptually distinct subscales: five adaptive strategies subscales (acceptance, positive refocusing, refocusing on planning, positive reappraisal, and putting into perspective) and four maladaptive strategies subscales (self-blame, rumination, catastrophizing, and blaming others) [22]. Four items are used to evaluate each of the cognitive emotion regulating strategies measured on a 5-point Likert scale, ranging from almost never (1) to almost always (5). Subscale scores are obtained by summing the scores for items in particular subscales (range, 4–20), with a higher subscale score indicating greater use of a specific cognitive strategy. Both the original version and Spanish CERQ have been found to have satisfactory internal consistencies and validity in numerous populations [23–37].

### Other data

We collected the socio-demographic information on the study sample using a socio-demographic data sheet which included age, gender, marital status, educational level, and length of unemployment.

### Statistical analyses

Data were analyzed using Statistical Package for the Social Sciences, version 20.0. Descriptive statistics were used to characterize the study sample in terms of socio-demographic variables. Because of previously reported gender differences on the CERQ [38], a one-way (gender) Multivariate Analysis of Variance (MANOVA) was computed on cognitive emotion regulation strategies. When the MANOVA yielded a significant main effect, subsequent univariate F-tests were performed. The level of significance was determined with p < 0.05.

To examine the associations of cognitive emotion regulation strategies with HRQoL, two hierarchical multiple regressions were conducted to determine the best set of predictors for MCS and PCS adjusting for the mutual correlations among CERQ scales. The regression analysis was performed in two steps. In the first step, background variables including gender, age, educational level and length of unemployment were entered in order to control for their potential effect on HRQoL. In the second step, the nine CERQ scales were entered. To examine whether coping strategies accounted for a small, medium, or large amount of the variance in HRQoL, we used Cohen’s convention for small (*f* ^*2*^ = .02), medium (*f* ^*2*^ = .15), and large effects (*f* ^*2*^ = .35) [39]. Two indicators, the tolerance and the Variance inflation factor, were used to detect multicollinearity between the predictor variables. A tolerance of less than 0.10 and/or a variance inflation factor of more than 10 indicate a multicollinearity problem [40].

## Results

### Descriptive analyses

Means, standard deviations, and Cronbach’s alpha coefficients for the study variables are shown in Table 2. The cognitive emotion regulation strategy most used in the total sample was refocusing planning and the least used was catastrophizing.

**Table 2:** **Gender differences between unemployed males and females in the use of different cognitive emotion regulation strategies and SF-12 scores: Means, Standard Deviations and F-test**

	**Total**	**Female**	**Male**	
	**N = 1125**	**N = 619**	**N = 506**	
	**M(SD)**	**M(SD)**	**M(SD)**	***p***
Self-blame	2.31(.87)	2.24(.87)	2.39(.86)	.00
Acceptance	3.00(.88)	2.98(.89)	3.03(.87)	.34
Rumination	2.82(.90)	2.80(.86)	2.85(.95)	.35
Positive Refocusing	3.17(.95)	3.20(.95)	3.13(.96)	.19
Refocus on Planning	3.42(.90)	3.44(.89)	3.40(.93)	.38
Positive Reappraisal	3.29(.99)	3.34(.98)	3.24(1.00)	.12
Putting into Perspective	3.33(.92)	3.38(.91)	3.27(.94)	.04
Catastrophizing	2.28(.92)	2.18(.90)	2.40(.93)	.00
Other-blame	2.57(1.11)	2.47(1.13)	2.70(1.07)	.00
Mental Composite Summary	46,33(10.79)	45.49(10.99)	47.35(10.47)	.00
Physical Composite Summary	51,36(7.83)	50.97(8.09)	51.84(7.49)	.06

As can be seen in Table 2, results showed that there were significant overall differences between males and females (*Wilks*’ *lambda* = .970; F (9, 1100) = 3,78; p = .000; η^2^ = .030). Univariate F-test showed that the significant differences were in self-blame (F(1,1100) = 8.04, *p* < .01, η^2^ = .007), other-blame (F(1,1100) = 12.34, *p* < .01, η^2^ = .011), catastrophizing (F(1, 1100) = 15.31, *p* < .01, η^2^ = .014), and perspective taking (F(1,1100) = 3.99, *p* < .05, η^2^ = .004). Specifically, men tended to express greater use of self-blame, other-blame, and catastrophizing and lower use of perspective taking strategies related to the experience of unemployment in their lives. Finally, univariate ANOVA analyzes revealed significant gender differences for MCS (F(1,1124) = 8,25; p < 0.01,) and marginally significant for PCS (F(1,1124) = 3,42; p = 0.06) with males obtaining higher scores than females in both cases. Accordingly, gender was entered into the subsequent partial correlation and hierarchical regression analyses as a control variable.

### Partial correlation analyses

Then, we examined the partial correlations among cognitive emotion regulation strategies, and mental and physical quality of life scores from SF-12 adjusting for gender. MCS was significantly and negatively related to self-blame, blaming others, rumination, and catastrophizing and positively associated to positive reappraisal, putting into perspective, planning, and positive refocusing. Regarding PCS, higher scores in physical health were significantly and negatively associated with blaming others, rumination, and catastrophizing and positively related to putting into perspective, positive reappraisal, planning, and positive refocusing.

### Hierarchical regression analyses

Finally, two hierarchical multiple regressions were conducted to determine the best set of predictors for both MCS and PCS scores. Gender, age, educational level and length of unemployment were entered in Step 1 to control for its confounding effects, and the nine CERQ subscales were entered simultaneously in Step 2 (see Table 3). In both regression equations multicollinearity did not appear to be a problem for the analysis (variance inflation factor <2.30; tolerance ranging from .43 to .96).

**Table 3:** **Hierarchical multiple regression predicting Mental and Physical Composite Summary accounted for by different cognitive emotion regulation strategies**

	**R** ^**2**^	**F**	**Unstandardized coefficients**		**Standardized coefficients**	**95% Confidence Interval for B**		**ΔR** ^**2**^
***Mental Composite Summary***			**B**	**Standard error**	**β**	**Lower limit**	**Upper limit**	
*Covariates*	0.01	2.77						0.01*
Sex			−2.43	.61	-.11**	−3.65	−1.22	
Age			-.03	.02	-.03	-.08	.02	
Educational level			-.62	.20	-.08**	−1.02	-.22	
Length of unemployment			.00	.01	.02	-.01	.02	
*Cognitive emotion regulation strategies*	0.17	17.79						0.17**
Self-blame			-.98	.41	-.08*	−1.78	-.17	
Acceptance			-.29	.41	-.02	−1.11	.52	
Rumination			−1.96	.42	-.16**	−2.80	−1.13	
Positive Refocusing			2.25	.41	.20**	1.45	3.06	
Refocus on Planning			-.65	.48	-.05	−1.55	.28	
Positive Reappraisal			2.45	.46	.22**	1.55	3.36	
Putting into Perspective			-.31	.43	-.02	−1.17	.54	
Catastrophizing			−1.73	.41	-.15**	−2.55	-.91	
Other-blame			.02	.30	.00	-.58	.62	
***Physical Composite Summary***								
*Covariates*	0.08	23.11						0.08**
Sex			−1.44	.45	-.07**	−2.34	-.54	
Age			-.13	.02	-.20**	-.17	-.09	
Educational level			.17	.76	.09**	.17	.76	
Length of unemployment			-.00	.00	-.03	-.02	.00	
*Cognitive emotion regulation strategies*	0.14	13.32						0.14**
Self-blame			.23	.30	.02	-.36	.82	
Acceptance			-.57	.31	-.06	−1.18	.03	
Rumination			-.89	.31	-.09*	−1.51	-.27	
Positive Refocusing			.03	.30	-.00	-.56	.62	
Refocus on Planning			1.82	.35	.21**	1.12	2.52	
Positive Reappraisal			-.32	.34	-.02	−1.00	.34	
Putting into Perspective			.05	.32	.00	-.58	.68	
Catastrophizing			−1.09	.31	-.15**	−1.70	-.48	
Other-blame			-.24	.22	-.04	-.69	.20	

Note: **p < 0.01; *p < 0.05.

As seen in Table 3, with regard to MCS, a total of 17% of this variance was accounted for (R = 0.46, R^2^ = 0.17; F(13,1085) = 17.79; *p* < 0.001), with gender accounting for a small (*f*^2^ = .01) but significant 1% of variance (*p* < 0.05) and cognitive emotion regulation strategies were found to account for a medium (*f*^2^ = .19) and significant variance 16% (*p* < 0.01) in MCS. In short, independent of gender, unemployed individuals who showed lower scores of self-blame, rumination, and catastrophizing, as well as higher scores of positive reappraisal and positive refocusing obtained higher scores on MCS.

For PCS, a total of 14% of this variance was accounted for (R = 0.37, R^2^ = 0.14; F(13, 1085) = 13.32; *p* < 0.001), with age accounting for a small (*f*^2^ = .06) but significant 6% of unique variance (b = −0.22, *p* < 0.01) and cognitive emotion regulation strategies were found to account for a small (*f*^2^ = 07.) but significant 7% of additional variance in predicting PCS. In short, independent of age, unemployed individuals who showed lower scores of catastrophizing and rumination and high scores in refocusing planning obtained higher scores on PCS.

## Discussion

The present study is the first to report associations between cognitive emotion regulation strategies and HRQoL in a relatively large sample of unemployed adults. The present findings generally confirm previously reported relationships between cognitive emotion regulation strategies from CERQ subscales and indicators of mental health in different populations [20,23–27]. Furthermore, our present findings also support the utility of the cognitive emotion regulation strategies in predicting physical health in the unemployed; a variable in which the potential utility of the CERQ had not previously been assessed.

First, data from gender differences revealed that unemployed men tended to express greater use of maladaptive strategies such as self-blame, other-blame, and catastrophizing and lower use of perspective taking strategies related to the experience of unemployment in their lives. These data are in line with previous findings that have showed the use of negative coping of unemployment in men [41]. Accordingly, our results suggest that, beyond societal norms and expectations which play an important role in how men and women will experience unemployment [42], another plausible explanation on the gender differences associated with the adverse effect of unemployment on well-being, might be the cognitive strategies used differently by men and women in response to the loss of their job. Therefore, since these coping strategies tend to be amenable to a psychosocial approach and might thereby increase quality of life for the unemployed, a program of further interventions aimed at teaching problem solving skills in job seeking and improving HRQoL in unemployed men should also aim to modify certain maladaptive cognitive emotion regulation strategies. Our findings give credibility to psychotherapeutic interventions using cognitive behavioral therapy which view the increased use of adaptative coping with job loss as a key goal for the effectiveness of the therapeutic process in terms of decreased symptoms of psychological distress [43] and higher rates of re-employment [44].

Second, our findings from correlations analyses suggest that individual differences in the propensity to experience lower scores in MCS among unemployed people were associated with high self-blame, blaming others, rumination, and catastrophizing and low positive reappraisal, putting into perspective, planning, and positive refocusing. These findings support the prominent role of these coping processes in cognitive theories of emotional distress [18,45]. Moreover, regression analyses on MCS, once controlled for socio-demographics, and all strategies entered simultaneously, show that better predictors of high MCS were lower scores of self-blame, rumination, and catastrophizing, as well as higher scores of positive reappraisal and positive refocusing. The findings suggest that strong self-blame, rumination, and catastrophizing as well as low reported use of positive reappraisal and positive refocusing during a period of unemployment should be considered as maladaptive cognitive emotion regulation strategies. From theoretical models of coping with job loss, unemployment seems to be a stressor to which people do not generally habituate. As people are confronted with prolonged stress caused by unemployment, they may experience a greater need for coping strategies that manage negative emotional intensity. According to our results, some cognitive regulation strategies might be more adaptive in terms of increased HRQoL. Our findings fit in with prior work in the general population and other potentially stressed samples where the same strategies were related to maladjustment [27]. Remarkably, independent of respondent gender, and with the exception of acceptance, all strategies were associated with mental health supporting the validity of the CERQ as a measure of cognitive coping styles useful for predicting variation in the HRQoL of the unemployed.

The present findings also examined the utility of the CERQ in predicting physical health outcomes in the unemployed. Independent of gender, age, educational level and length of unemployment, physical health was predicted by lower scores of catastrophizing and rumination and high scores in planning. Previous theoretical justification for research into coping underlines that those who engage in adaptive cognitive coping strategies will experience greater positive health effects on self-perceived physical health than those who engage in maladaptive cognitive coping [46,47]. Our findings are in accordance with this assertion and show, specifically, which cognitive coping strategies appear to be better predictors with regard to physical health associated with being unemployed.

Among the limitations to be considered in this study, it is important to highlight the cross-sectional design; therefore, direct causal statements cannot be made with certainty. Further research should examine questions such as whether the use of cognitive emotion regulation strategies by the unemployed refers to a stable pattern, or whether the profile of coping strategies employed by an individual may change during the course of unemployment. That is, a long duration of unemployment may provoke appraisals that job loss is irreversible, which may promote maladaptive coping and decrease adaptive coping in unemployed individuals. A closer examination of the nature of this relationship should be performed using longitudinal studies. Thus, our study included unemployed individuals recruited by purposive sampling (snowball techniques), which is a non-random sampling technique. Snowball samples may be more biased toward the more cooperative unemployed participants who are willing to participate in the study which limits the generalization of our results. Besides, although the study sample was representative in terms of gender of the unemployed in Spain area, our study sample was older than National statistics Institute [1], a finding that restricts the external validity of the results. Another limitation of the design was that the assessments of cognitive emotion regulation strategies and HRQoL were both based on a self-report which may have caused some bias such as overestimation (or underestimation) and inherent shared method variance between the measures. Future studies should also include other measurement methodologies, such as focus group interview data, situational judgment test, expert judgments or clinical diagnosis. Generalizing the findings beyond the current sample would only be possible after replicating the study with other random unemployed populations, with complementary measurement methodologies, and including prospective elements to enable conclusions to be drawn about the causality between cognitive coping patterns used at an early stage of unemployment and subsequent HRQoL levels.

### Implications

In sum, independent of gender, educational level and length of unemployment, the use of cognitive emotion regulation strategies seems to explain a unique and significant part of the variation in unemployed HRQoL. These coping strategies tend to be amenable to a psychosocial approach and can thereby increase an unemployed individual’s medium and long-term quality of life. If these findings can be replicated, then it might be worthwhile designing occupational interventions that encourage unemployed individuals to use adaptive (i.e. positive refocusing, refocusing planning) rather than maladaptive cognitive emotion regulation strategies (i.e. self-blame, blaming others, rumination, and catastrophizing), as is offered in other intervention program designs for other samples for a diverse range of stressful experiences [48–50]. Furthermore, our findings suggest that cognitive coping patterns and emotion regulation strategies should be explored, evaluated, and discussed with the unemployed, and, if necessary, corrected at an early stage. Adaptive emotion regulation strategies should be stimulated, and negative coping patterns should be prevented or challenged over the unemployment period. These cognitive coping approaches may result in better HRQoL in the unemployed during their job search process. Finally, since intervention programs focusing on cognitive emotion regulation factors have only recently been described in populations with diverse chronic strains [48–50], it is necessary for individual-level interventions to implement and examine the specific effectiveness of these prevention programs for unemployed people.

## Conclusions

Since the adverse impact of job loss on the unemployed individual goes beyond employment benefits and income loss, studies of antecedents and correlates of HRQoL may be of considerable significance in understanding unemployed HRQoL and its development. Also the use of coping mechanisms and the effects of these on daily life stress such as job rejections, financial adversity, interviews, amongst others should be further examined [5,6]. The present study showed that HRQoL is associated with several cognitive emotion regulation strategies. The high use of rumination, catastrophizing, and self-blame (for MCS only) were maladaptive strategies associated to reduced HRQoL. In addition, higher scores of positive reappraisal, positive refocusing (for MCS), and planning (for PCS) were crucial strategies in sustaining better HRQoL and were considered adaptive coping strategies. Given that theorists argue that these coping strategies may be changed and learned through school programs or experience, our preliminary findings might serve as a good starting point for inclusion of cognitive coping as an additional intervention strategy to complement current vocational training approaches to improve the health of unemployed people. The practical applications of these results in the area of health services and occupational counselling indicate that those unemployed people with higher use of maladaptive coping strategies during periods of unemployment may be targeted for intervention to develop better adaptive cognitive emotional regulation for managing the stress of job loss, directed toward increasing HRQoL. Undoubtedly, a better understanding of the underlying cognitive mechanisms in people when confronted with unemployment will promote the early identification of psychological distress and the implementation of appropriate preventive interventions, facilitating better adjustment to unemployment.

## Abbreviations

HRQoL: Health-related quality of life
SF-12: 12-Item short form survey
MCS: Mental component summary
PCS: Physical component summary
CERQ: Cognitive emotion regulation questionnaire
MANOVA: Multivariate analysis of variance

## References

[1] INE. Instituto Nacional de Estadística [National Statistics Institute]. [cited 2014 16/05/2014]. Available at: http://www.ine.es/

[2] McKee-Ryan F, Song Z, Wanberg CR, Kinicki AJ (2005). Psychological and physical well-being during unemployment: a meta-analytic study. J Appl Psychol.

[3] Murphy GC, Athanasou JA (1999). The effects of unemployment on mental health. J Occup Organ Psych.

[4] Stankunas M, Kalediene R, Starkuviene S, Kapustinskiene V (2006). Duration of unemployment and depression: a cross-sectional survey in Lithuania. BMC Public Health.

[5] Tiggemann M, Winefield AH (1984). The effects of unemployment on the mood, self-esteem, locus of control, and depressive affect of school-leavers. J Occup Psych.

[6] Preti A (2003). Unemployment and suicide. J Epidemiol Commun H.

[7] Lundin A, Hemmingsson T (2009). Unemployment and suicide. Lancet.

[8] Wanberg CR (2012). The individual experience of unemployment. Annu Rev Psychol.

[9] Paul KI, Moser K (2009). Unemployment impairs mental health: meta-analyses. J Vocat Behav.

[10] Langens TA, Mose E (2006). Coping with unemployment: relationships between duration of unemployment, coping styles, and subjective well-being. J Appl Biobehav Res.

[11] Grossi G (1999). Coping and emotional distress in a sample of Swedish unemployed. Scand J Psychol.

[12] Waters LE (2000). Coping with unemployment: a literature review and presentation of a new model. Int J Manag Rev.

[13] Higgins JE, Endler NS (1995). Coping, life stress, and psychological and somatic distress. Eur J Pers.

[14] Warr PB (1997). Work, unemployment and mental health.

[15] Leana CR, Feldman DC (1992). Coping with job loss: How individuals, organizations, and communities respond to layoffs.

[16] Latack JC, Havlovic SJ (1992). Coping with job stress: a conceptual evaluation framework for coping measures. J Organ Behav.

[17] Lazarus RS (1999). Stress and emotion: a new synthesis.

[18] Lazarus RS, Folkman S (1984). Stress, appraisal, and coping.

[19] Compas BE, Connor-Smith JK, Saltzman H, Harding-Thomsen A, Wadsworth ME (2001). Coping with stress during childhood and adolescence: problems, progress, and potential in theory and research. Psychol Bull.

[20] Garnefski N, Kraaij V, Spinhoven P (2001). Negative life events, cognitive emotion regulation and depression. Pers Indiv Differ.

[21] Garnefski N, Kraaij V, Spinhoven P (2002). Manual for the use of the cognitive emotion regulation questionnaire.

[22] Thompson RA (1991). Emotional regulation and emotional development. Educ Psychol Rev.

[23] Garnefski N, Kraaij V (2007). The cognitive emotion regulation questionnaire: psychometric features and prospective relationships with depression and anxiety in adults. Eur J Psychol Assess.

[24] Martin RC, Dahlen ER (2005). Cognitive emotion regulation and the prediction of depression, anxiety, stress, and anger. Pers Indiv Differ.

[25] Schroevers MJ, Kraaij V, Garnefski N (2007). Goal disturbance, cognitive coping strtegies, and psychological adjustment to different types of stressful life event. Pers Indiv Differ.

[26] Garnefski N, Legerstee J, Kraaij V, Van den Kommer T, Teerds J (2002). Cognitive coping strategies and symptoms of depression and anxiety: a comparison between adolescents and adults. J Adolescence.

[27] Garnefski N, Kraaij V (2006). Relationships between cognitive emotion regulation strategies and depressive symptoms: a comparative study of five specific samples. Pers Indiv Differ.

[28] Kraaij V, Pruymboom E, Garnefski N (2002). Cognitive coping and depressive symptoms in the elderly: a longitudinal study. Aging Ment Health.

[29] Wang Y, Yi J, He J, Chen G, Li L, Yang Y, Zhu X (2014). Cognitive emotion regulation strategies as predictors of depressive symptoms in women newly diagnosed with breast cancer. Psycho-Oncol.

[30] Endler NS, Parker JDA (1994). Assessment of multidimensional coping. Psychol Assess.

[31] EuroStat (2011). EuroStat Database.

[32] WHO-Europe (2009). Health in Times of Global Economic Crisis: Implications for the WHO European Region.

[33] Dooley D, Fielding J, Levi L (1996). Health and unemployment. Annu Rev Publ Health.

[34] Ware JE, Kosinski M, Keller SD (1996). A 12-item short-form health survey: construction of scales and preliminary tests of reliability and validity. Med Care.

[35] Vilagut G, Valderas JM, Ferrer M, Garin O, López-García E, Alonso J (2008). Interpretación de los cuestionarios de salud SF-36 y SF-12 en España: componentes físico y mental. Med Clin-Barcelona.

[36] Hendricks VM, Blanken P, Hendricks VM, Blanken P, Adriaans N (1992). Snowball sampling: theoretical and practical considerations. Snowball sampling: a pilot study on cocaine use.

[37] Domínguez-Sánchez FJ, Lasa-Aristu A, Amor PJ, Holgado-Tello FP (2013). Psychometric properties of the Spanish version of the cognitive emotion regulation questionnaire. Assessment.

[38] Garnefski N, Teerds J, Kraaij V, Legerstee J, Van den Kommer T (2004). Cognitive emotion regulation strategies and depressive symptoms: differences between males and females. Pers Indiv Differ.

[39] Cohen J (1988). Statistical power analysis for the behavioral sciences.

[40] O’Brien RM (2007). A caution regarding rule of thumb for variance inflation factors. Qual Quant.

[41] Lee C, Owens RG (2002). Men, work, and gender. Aust Psychologist.

[42] Phillips SD, Imhoff AR (1997). Women and career development: a decade of research. Annu Rev Psychol.

[43] Creed PA, Machin MA, Hicks RE (1999). Improving mental health status and coping abilities for long-term unemployed youth using cognitive-behaviour therapy based training interventions. J Organ Psych.

[44] Vuori J, Silvonen J (2005). The benefits of a preventive job search program on re-employment and mental health at 2-year follow-up. J Occup Organ Psych.

[45] Beck AT (1975). Cognitive therapy and the emotional disorders.

[46] Aldwin CM, Park CL (2004). Coping and physical health: an overview. Psychol Health.

[47] Penley JA, Tomaka J, Wiebe JS (2002). The association of coping with physical and mental health outcome: a meta-analytic review. J Behav Med.

[48] Garnefski N, Kraaij V, Wijers E, Hamming J (2013). Effects of a cognitive-behavioral self-help program on depressed mood for people with peripheral arterial disease. J Clin Psychol Med S.

[49] Garnefski N, Kraaij V (2012). Effects of a cognitive behavioral self-help program on emotional problems for people with acquired hearing loss: a randomized controlled trial. J Deaf Stud Deaf Educ.

[50] Garnefski N, Kraaij V, De Graaf M, Karels L (2010). Psychological intervention targets for people with visual impairments: the importance of cognitive coping and goal adjustment. Disabil Rehabil.

